# Let‐7a promotes periodontal bone regeneration of bone marrow mesenchymal stem cell aggregates via the Fas/FasL‐autophagy pathway

**DOI:** 10.1111/jcmm.17988

**Published:** 2023-10-19

**Authors:** Shiyao Yang, Jing Gao, Meng Chen, Yuting Sun, Xin Qiao, Hongchen Mao, Li Guo, Yang Yu, Deqin Yang

**Affiliations:** ^1^ Department of Endodontics Stomatological Hospital of Chongqing Medical University Chongqing China; ^2^ Stomatological Hospital of Chongqing Medical University Chongqing Key Laboratory of Oral Diseases and Biomedical Sciences Chongqing China; ^3^ Chongqing Municipal Key Laboratory of Oral Biomedical Engineering of Higher Education Chongqing China; ^4^ Department of Oral and Maxillofacial Surgery Daping Hospital, Army Medical University Chongqing China; ^5^ Chongqing Key Laboratory of Oral Diseases and Biomedical Sciences Chongqing China

**Keywords:** BMMSCs, cell‐aggregates, Fas/FasL, microRNA, periodontal regeneration

## Abstract

Periodontal bone regeneration using bone marrow mesenchymal stem cell (BMMSC) transplantation is a promising method; however, the method for osteogenic differentiation of BMMSCs needs to be improved. In this research, we sought to identify the roles of let‐7a in the osteogenesis of BMMSCs and to provide a potential method for periodontal bone regeneration. Our previous study revealed that Fas/FasL is a target of let‐7a. In this study, we demonstrated that let‐7a overexpression significantly enhanced BMMSC‐CAs osteogenesis both in vitro and in vivo. Mechanistically, upregulation of Fas/FasL using the *rfas*/*rfaslg* plasmid obstructed the osteogenesis of BMMSCs by inhibiting autophagy. Furthermore, we confirmed that overexpression of let‐7a activated autophagy and alleviated the inhibited osteogenesis by the autophagy inhibitor 3‐MA and the *rfas*/*rfaslg* plasmid of BMMSCs. In general, our findings showed that let‐7a promoted the osteogenesis of BMMSCs through the Fas/FasL‐autophagy pathway, suggesting that the application of let‐7a in BMMSC‐CAs based periodontal bone regeneration could be a promising strategy.

## 1 INTRODUCTION

For periodontal bone regeneration, tissue engineering based on stem cells has been introduced.^1^ As a type of mesenchymal stem cells (MSCs), bone marrow mesenchymal stem cells (BMMSCs) with self‐renewal and multipotent differentiation capacities^2^ have become the predominant seed cells used for bone tissue repair.^3^, ^4^ Although numerous animal experiments have revealed that BMMSCs repair damaged periodontal bone tissue,^5^, ^6^, ^7^ the effects are still limited. Recently, cell aggregate (CA) technology has become a scaffold‐free strategy for tissue regeneration that can protect cells from being damaged by enzymes so that the extracellular matrix (ECM) secreted by BMMSCs remains intact.^8^ Recent research has increasingly concentrated on the role of MSC‐CAs in periodontal regeneration.^9^ Thus, it is crucial to explore how to promote BMMSC‐CA osteogenesis for periodontal bone regeneration.

Small endogenous noncoding RNAs named microRNAs (miRNAs) target mRNAs post‐transcriptionally by binding to complementary regions.^10^ Several investigations have demonstrated the importance of miRNAs for regulating MSC osteogenesis.^11^ For example, Lin et al. showed that miR‐130a enhances BMSC differentiation into osteoblasts by inhibiting the expression of Smurf2.^12^ According to other researchers, the Smad7‐Smad1/5/8‐Runx2 pathway enhances the osteogenesis of BMMSCs by miR‐21.^13^ Targeting miRNAs was shown to be a viable strategy for bone tissue regeneration.^14^ Among these miRNAs, miRNA‐let‐7a, a member of the let‐7 family, is one of the most conserved miRNAs^15^, ^16^ and is highly expressed in MSCs.^17^ Research has shown that let‐7 can positively regulate bone formation and osteogenesis.^18^ Moreover, let‐7 is upregulated during human MSC osteogenic differentiation.^19^, ^20^ Subsequent studies revealed that let‐7a is a crucial regulator of cell growth, differentiation, and proliferation.^21^ Therefore, let‐7a might be important in the differentiation of BMMSCs toward osteoblasts. As a sheet of interconnected BMMSCs,^9^ whether let‐7a could promote the osteogenesis of BMMSC‐CAs has not been studied.

Osteogenic differentiation is an intricate process regulated by several signalling pathways.^22^ Our group has reported that let‐7a targets Fas/FasL pathways and inhibits the expression of the Fas and FasL proteins.^17^ The Fas/FasL signalling pathway is crucial for cell differentiation, apoptosis^23^ and bone homeostasis.^24^, ^25^ Fas can be found in a wide range of cells, and the binding of Fas to its ligand FasL is important in the regulation of osteogenesis.^26^, ^27^ As a classical apoptosis pathway, the regulation of the Fas/FasL pathway influences autophagy,^28^, ^29^ and autophagy is essential for regulating the osteogenesis of BMMSCs and is also a key regulator of bone metabolism.^30^, ^31^ Therefore, we investigated whether let‐7a could enhance the autophagy of BMMSCs by Fas/FasL and then accelerate the bone tissue defect repair of BMMSC‐CAs. In this research, we proved that let‐7a enhanced the osteogenesis of BMMSC‐CAs via the Fas/FasL‐autophagy pathway in vitro and evaluated the effects of let‐7a on BMMSC‐CA bone regeneration ability in vivo, suggesting the potential of let‐7a in BMMSC‐CA‐based periodontal bone tissue repair in the clinic.

## 2 MATERIALS AND METHODS

### 2.1 Animals

Sprague–Dawley (SD) rats (6 weeks) and nude mice (6 weeks) were purchased from the Laboratory Animal Center of Chongqing Medical University. The Animal Care and Use Committee of Chongqing Medical University authorized all animal research carried out in accordance with the National Institutes of Health [Ethical code: 2020(067)].

### 2.2 Cell culture

BMMSCs were obtained as previously described with a few modest modifications.^32^ Briefly, SD rats were used to harvest BMMSCs using complete medium [α‐MEM (Invitrogen) containing 10% fetal bovine serum (FBS) (Sijiqing) and 1% penicillin and streptomycin (Sigma‐Aldrich)]. Then, the cells were placed in a humidified environment at 37°C with 5% CO_2_ for incubation. BMMSCs were passaged when the confluence was 70%–80%. The following experiments were performed using P3 BMMSCs.

### 2.3 Colony‐forming unit (CFU) assays

Approximately 1 × 10^3^ BMMSCs (P3) were plated in a complete medium‐filled 10 cm culture dish for CFU assays. On Day 14, the cells underwent fixation using 4% paraformaldehyde and were stained with 1% crystal violet as previously reported.^9^

### 2.4 Cell proliferation assay

Cell Counting Kit‐8 (CCK‐8) assays (Dojindo) were used following the manufacturer's instructions. With a density of 2.5 × 10^3^ cells per well, 96‐well plates were utilized for seeding BMMSCs. Each well contained 90 μL of basal media and 10 μL of CCK‐8 reagent. Following a 2‐h incubation period at 37°C, the cells were evaluated for absorbance at 450 nm. BMMSCs were cultivated for 7 days, and on the first, third, fifth and seventh days, the OD value was determined.

### 2.5 Flow cytometry analysis

Briefly, after being washed twice with PBS (Biosharp) and detached with 1 mL of 0.25% trypsin (Mengbio), adherent cells at a density of 1 × 10^6^ were harvested. Then, BMMSCs were resuspended. The cells were incubated with rat CD29 (PE), CD90 (FITC), CD31 (PE) and CD45 (PE) (BD Bioscience) antibodies at 4°C in the dark. Finally, the samples were assessed using a flow cytometer.

### 2.6 Multiple differentiation assay

BMMSCs were plated at a density of 2 × 10^5^ cells per well with basal medium. When the cells were between 70% and 80% confluent, BMMSCs were induced with a rat BMMSC osteogenic induction kit for 4 weeks or adipogenic induction kit for 3 weeks based on the directions provided by the manufacturer (Oricell). Every 3 days, the medium was replaced. The BMMSCs were subsequently stained with 2% Alizarin Red S (osteogenic differentiation) or 0.3% oil red O (adipogenic differentiation) after being fixed with 4% paraformaldehyde.

### 2.7 Transfection of let‐7a

BMMSCs were transfected with let‐7a mimics, let‐7a inhibitor, and let‐7a negative control in accordance with the manufacturer's instructions (RiboBio). We bought let‐7a mimics, inhibitor, and negative control from RiboBio. With the use of Lipofectamine 2000 (Invitrogen), let‐7a was transfected. The final concentration of let‐7a inhibitor and negative control was 150 nM, and that of the mimics was 50 nM according to previous reports.^17^

### 2.8 Construction and transfection of the plasmid

The Fas/FasL overexpression vector was synthesized by RiboBio. The let‐7a mimics group, *rFas*/*rFaslg* group, let‐7a mimics + *rFas*/*rFaslg* group and control group were the various transfection groups. With Lipofectamine 2000 (Invitrogen), transfection was performed in accordance with the manufacturer's instructions. In brief, BMMSCs were digested, suspended, counted, and then plated into 24‐well plate at a density of 1 × 10^5^ cells/well. After 6–8 h of incubation, the medium was replaced into complete medium without penicillin and streptomycin (α‐MEM containing 10% FBS). Then, we diluted the plasmid and Lipofectamine 2000 with α‐MEM respectively, and finally, we mixed them and added them alone or with let‐7a mimics into a 24‐well plate.

### 2.9 Reverse transcription quantitative polymerase chain reaction (RT–qPCR)

TRIzol reagent (Invitrogen) was used to extract total RNA from BMMSCs. With a TaKaRa PrimeScript RT Reagent Kit from TaKaRa, cDNA was produced by reverse transcription of RNA. The reverse‐transcribed cDNA products were diluted with 10 μL of DEPC water. Followed‐up PCR reaction was conducted by a SYBR® Premix Ex TaqTM (Perfect Real Time) kit (TaKaRa). The following settings were used to conduct the reactions: 95°C for 15 s (40 cycles), 60°C for 35 s; 72°C for 30 s; 65°C for 15 s; 95°Cfor 0 s, 0.5°C/s. Table 1 lists the amplification primer sequences.

**TABLE 1:** Primer sequences for real time‐polymerase chain reaction.

Gene	Gene primer sequence
ALP	F 5′‐GGACCATTCCCACGTCTTCAC‐3′ R 5′‐CCTTGTAGCCAGGCCCATTG‐3′
RUNX2	F 5′‐GCACCCAGCCCATAATAGA‐3′ R 5′‐TTGGAGCAAGGAGAACCC‐3′
OSX	F 5′‐GCCTACTTACCCGTCTGACTTT‐3′ R 5′‐GCCCACTATTGCCAACTGC‐3′
Col‐1	F 5′‐TTCCCGGTGAATTCGGTCTC‐3′ R 5′‐ACCTCGGATTCCAATAGGACCAG‐3′
Fibronectin	F 5′‐CCAGTTAGGGTTGGCGTCTTC‐3′ R 5′‐GCTGGTCCATGCTCAGAGTGTC‐3′
Integrin β1	F 5′‐CCGCGCGGAAAAGATGAATTT‐3′ R 5′‐AGCAAACACACAGCAAACTGA‐3′
Beclin	F 5′‐CAGTACCAGCGGGAGTATAGTGA‐3′ R 5′‐TGTGGAAGGTGGCATTGAAGA‐3′
LC3	F 5′‐CCTGTCCTGGATAAGACCAAGTT‐3′ R 5′‐CTCCTGTTCATAGATGTCAGCGAT‐3′
Fas	F 5′‐TTCCCATCCTCCTGACCAC‐3′ R 5′‐CTCGTAAACCGCTTCCCTC‐3′
FasL	F 5′‐ATTGGCACCATCTTTACTTACC‐3′ R 5′‐CTCCTTAGAATCTGCTCTCATA‐3′
let‐7a	F 5′‐GTGTATCATACAGTATAATGAAACTAC‐3′ R 5′‐AACAGTGCAGTTAGTTCT‐3′
GAPDH	F 5′‐GGCACAGTCAAGGCTGAGAATG‐3′ R 5′‐ATGGTGGTGAAGACGCCAGTA‐3′

### 2.10 Western blot analysis

The total protein in the cells was extracted using RIPA lysis buffer, and the concentration of the protein sample was determined using a BCA kit (Beyotime Biotechnology). Each sample underwent a 10‐min denaturing process at 100°C. Samples were separated using a 10% SDS polyacrylamide gel (Beyotime Biotechnology) and transferred to PVDF membranes (Millipore). The membrane was first blocked with 5% nonfat milk TBST buffer for 2 h, and primary antibodies were applied and incubated overnight at 4°C, followed by the application of secondary antibodies for 1 h. Blots were visualized using BeyoECL Moon (Beyotime Biotechnology). The antibodies used in this study included Fas (1:2000; R&D System), FasL (1:1000; Bioss), COL‐I (1:1000; Immunoway), integrin β1 (1:1000; Immunoway), Fibronectin (1:1000; Immunoway), RUNX2 (1:1000; Immunoway), Osterix (1:1000; Affinity), ALP (1:2000; Santa Cruz Biotechnology), LC3B (1:1000; Abcam), Beclin (1:2000; Santa Cruz Biotechnology), HRP‐conjugated secondary antibodies against rabbit and mouse (1:10000; Biosharp), and HRP‐labelled donkey anti‐goat IgG (1:1000; Beyotime).

### 2.11 Construction of CAs

CAs were constructed as previously described.^9^ Briefly, BMMSCs were cultivated in basal media until they reached 70%–80% confluence after being plated at a density of 5 × 10^5^ cells/well in 6‐well plates. Then, the cells were induced with basal medium with 50 μg/mL vitamin C (VC), and the medium was replaced every 3 days. White membranous structures were observed at Day 10, and CAs thickened with time.

### 2.12 In vitro osteogenic assay

A BCIP/NBT ALP kit (Beyotime) was used to stain BMMSCs after 7 days of osteogenic incubation. An alkaline phosphatase assay kit (Nanjing Jiancheng Bioengineering) was used to evaluate ALP activity. BMMSCs were fixed with 4% paraformaldehyde and stained with 2% Alizarin Red S following 4 weeks of osteogenic induction. The calcium nodules were dissolved in 10% cetylpyridinium chloride solution for 1 h (Rhawn) as previously reported,^9^ and the absorbance was measured at 560 nm.

### 2.13 Subcutaneous transplantation in nude mice

After being mixed with 30 mg of hydroxyapatite tricalcium phosphate (HA‐TCP), the BMMSC‐CAs from each group were subcutaneously implanted into the dorsal area of nude mice. General anaesthesia was used during all procedures with a rodent anaesthesia machine. The mice were sacrificed 4 weeks post‐surgery. The extracted samples were stained with haematoxylin and eosin after being fixed with 4% paraformaldehyde.

### 2.14 Periodontal bone defect model

BMMSCs‐CA were divided into the mimics group (let‐7a mimics‐transfected CAs + HA‐TCP), inhibitor group (let‐7a inhibitor‐transfected CAs + HA‐TCP), negative control group (let‐7a negative control‐transfected CAs + HA‐TCP), and control group. Under air anaesthetic, the subsequent surgical procedures were carried out. A rat model of periodontal bone defects was constructed according to earlier reports.^9^ In brief, the mandible was exposed by entering through the masseter muscle on the buccal surface. The alveolar bone and the cementum over the mandibular first molar was taken away to create a defect of roughly 2× 2 mm, and the BMMSCs‐CA of each group carried by HA‐TCP were implanted into the bone defect. Mandibular specimens were taken at 4 and 6 weeks following surgery, examined using micro‐CT, and then stained with haematoxylin and eosin.

### 2.15 Immunofluorescence analysis

Immunofluorescence analysis was conducted as previously stated.^33^ Briefly, BMMSCs were plated in 12‐well plates at a density of 1 × 10^5^ cells per well following transfection, and they were subsequently treated for 4 h with 50 μM chloroquine (CQ) (Abmole) treatment for 4 h. After being fixed for 30 min with 4% paraformaldehyde in 12‐well plates, 0.03% Triton X‐100 was used to permeabilize the cells for 20 min. Following 3% BSA blocking of BMMSCs, the LC3 (1:100, Abcam) antibody was incubated overnight at 4°C. The anti‐rabbit secondary antibody was incubated for 1 h before DAPI (Biosharp) was used to counterstain the nuclei. With an inverted microscope, pictures were photographed.

### 2.16 Transmission electron microscopy (TEM)

Transfections of let‐7a mimics, inhibitor, and negative control were carried out on BMMSCs. After 24 h of transfection, cells were collected and fixed with 1% glutaraldehyde overnight. Samples were dehydrated, embedded, sectioned, and stained and then observed with TEM at an accelerating voltage of 80–120 kV.

### 2.17 Statistical analysis

GraphPad Prism 7.0 software was used to statistically evaluate all of the results. All values are described as the mean ± standard deviation (M ± SD). Student's *t*‐test was used to compare two independent samples. One‐way anova was used to determine statistical significance among three or more samples. *p* < 0.05 was deemed significant.

## 3 RESULTS

### 3.1 Let‐7a promotes ECM formation and osteogenesis of BMMSC‐CAs in vitro

**FIGURE 1:**
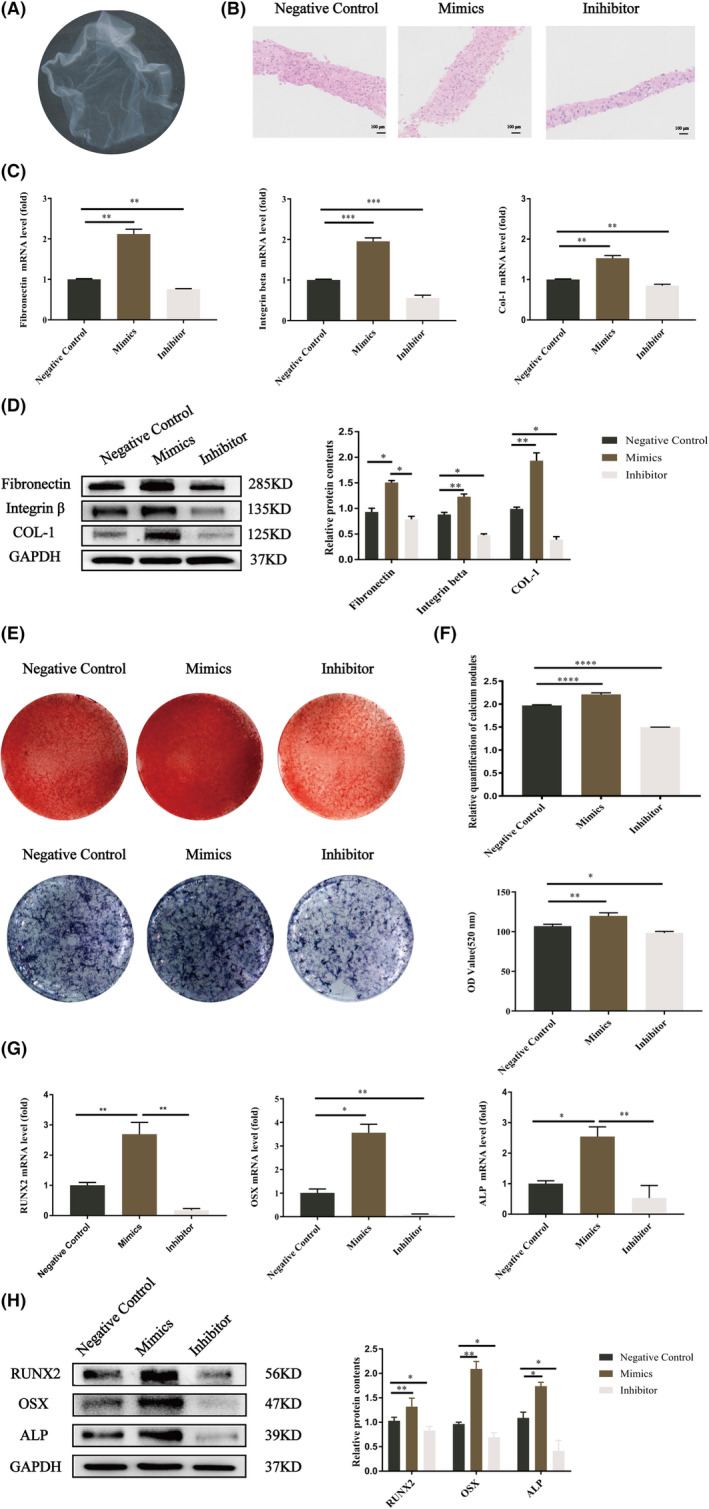
The effects of let‐7a transfection on the extracellular matrix formation and the osteogenic differentiation of BMMSC‐CAs in vitro. (A) Seven days after aggregation induction, macroscopic images observed of BMMSC cell‐aggregates plated on culture dishes. (B) Haematoxylin and eosin staining of BMMSC‐CAs of let‐7a mimics, inhibitor, negative control group (scale bar = 100 μm). (C) Relative mRNA levels of *Col*‐*1*, *Fibronectin* and *Integrin*‐*β* in BMMSC‐CAs of let‐7a mimics, inhibitor, negative control group. GAPDH was used for normalization. (D) Western blot of Col‐1, Fibronectin and Integrin‐β protein level in BMMSC‐CAs of each group after let‐7a transfection and quantification of band intensities. GAPDH was used as the internal control. (E) Alkaline phosphatase (ALP) staining and alizarin red S (ARS) staining of each group after let‐7a transfection. (F) Quantitative comparison of mineralized nodule formation and ALP activities of BMMSC‐CAs in each group after let‐7a transfection. (G) Relative mRNA levels of *ALP*, *RUNX2* and *OSX* in BMMSCs‐CA of let‐7a mimics, inhibitor, negative control group. *GAPDH* was used for normalization. (H)Western blot of ALP, RUNX2 and OSX protein level in BMMSC‐CAs of each group after let‐7a transfection and quantification of band intensities. GAPDH was used as the internal control. (Data are presented as means ± SEM, *n* = 3 independent experiments. *p < 0.05, **p < 0.01, ***p < 0.001).

BMMSCs were obtained and were identified by proliferation, multidirectional differentiation ability and flow cytometric analysis (Figure S1). We chose 24 h as the optimal transfection time because let‐7a mimics inhibited the expression of Fas/FasL the most (Figure S2). After induction with medium containing VC, BMMSC‐CAs could be harvested at the bottom of the well and showed a membrane‐like morphology with a certain thickness (Figure 1A). According to haematoxylin and eosin staining, the let‐7a mimics group produced the most ECM, while the let‐7a inhibitor group produced the least amount of ECM (Figure 1B). *Col*‐*1*, *integrin β*, and *Fibronectin* were all expressed at higher levels in the let‐7a overexpression group compared to the NC group but at lower levels in the let‐7a inhibitor group (Figure 1C,D). We next investigated the osteogenesis of BMMSC‐CAs transfected with let‐7a. ALP staining and activity, Alizarin Red staining and quantification showed that the let‐7a‐overexpressing BMMSC‐CAs displayed higher osteogenic differentiation than the let‐7a‐downregulated BMMSC‐CAs (Figure 1E,F). ALP, Runx2, and OSX had increased mRNA and protein levels in the let‐7a mimics group compared to the let‐7a NC group, while the let‐7a inhibitor group had lower levels (Figure 1G,H). In summary, BMMSC‐CAs may promote osteogenic differentiation and ECM formation by overexpressing let‐7a.

### 3.2 Let‐7a promotes the osteogenesis of BMMSC‐CAs *in vivo*

**FIGURE 2:**
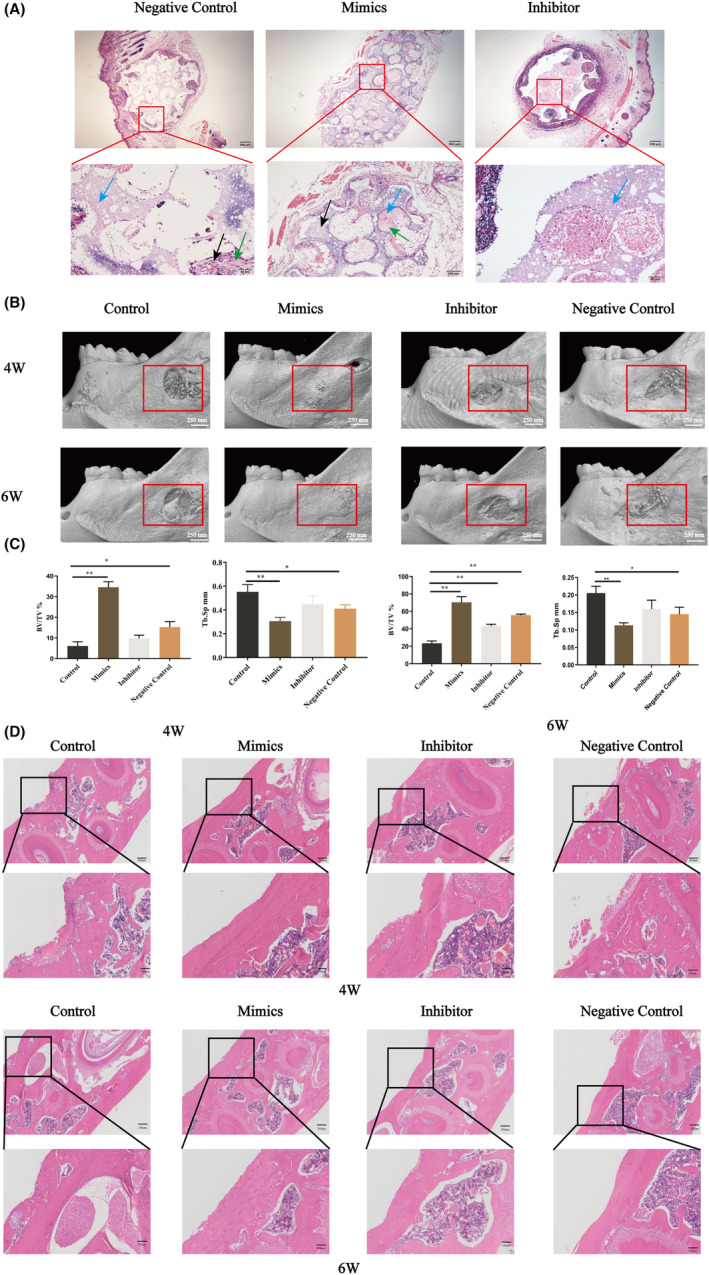
The effects of let‐7a transfection on the osteogenesis of BMMSC‐CAs of each group in vivo. (A) Haematoxylin and eosin staining of subcutaneous tissue after BMMSC‐CAs of let‐7a mimics, inhibitor and negative control group combined with HA‐TCP implanted into immunocompromised mice; black arrows: osteocytes and bone lacunae; green arrows: new born osteoid; blue arrows: HA‐TCP (scale bar = 200 μm or 50 μm). (B) Micro‐CT reconstruction was performed to detect the newly formed bone tissues in periodontal bone defect model in the 4th week and 6th week post‐surgery (scale bar = 250 mm). (C) The quantitative analysis of BV/TV and Tb.Sp of each group. (D) Haematoxylin and eosin staining of the mandibular specimens of each group. (Data are presented as means ± SEM, *n* = 3 independent experiments. *p < 0.05, **p < 0.01, ***p < 0.001).

Let‐7a could promote CA osteogenesis in vitro, and we further explored whether upregulation of let‐7a promotes bone formation of CAs in vivo. Haematoxylin and eosin staining results revealed that the let‐7a overexpression group formed the most osteocytes, bone lacunae and osteoids, but the let‐7a downregulated group formed the fewest (Figure 2A). The micro‐CT and quantitative results showed that the let‐7a‐upregulated BMMSC‐CAs produced more new bone tissue compared to the NC group, while the let‐7a inhibitor drastically reduced the fraction of newly produced bone tissue compared to the NC group (Figure 2B,C). The haematoxylin and eosin staining results of the mandible showed the same trend as the micro‐CT results (Figure 2D).

### 3.3 Let‐7a promotes the autophagy in BMMSCs

**FIGURE 3:**
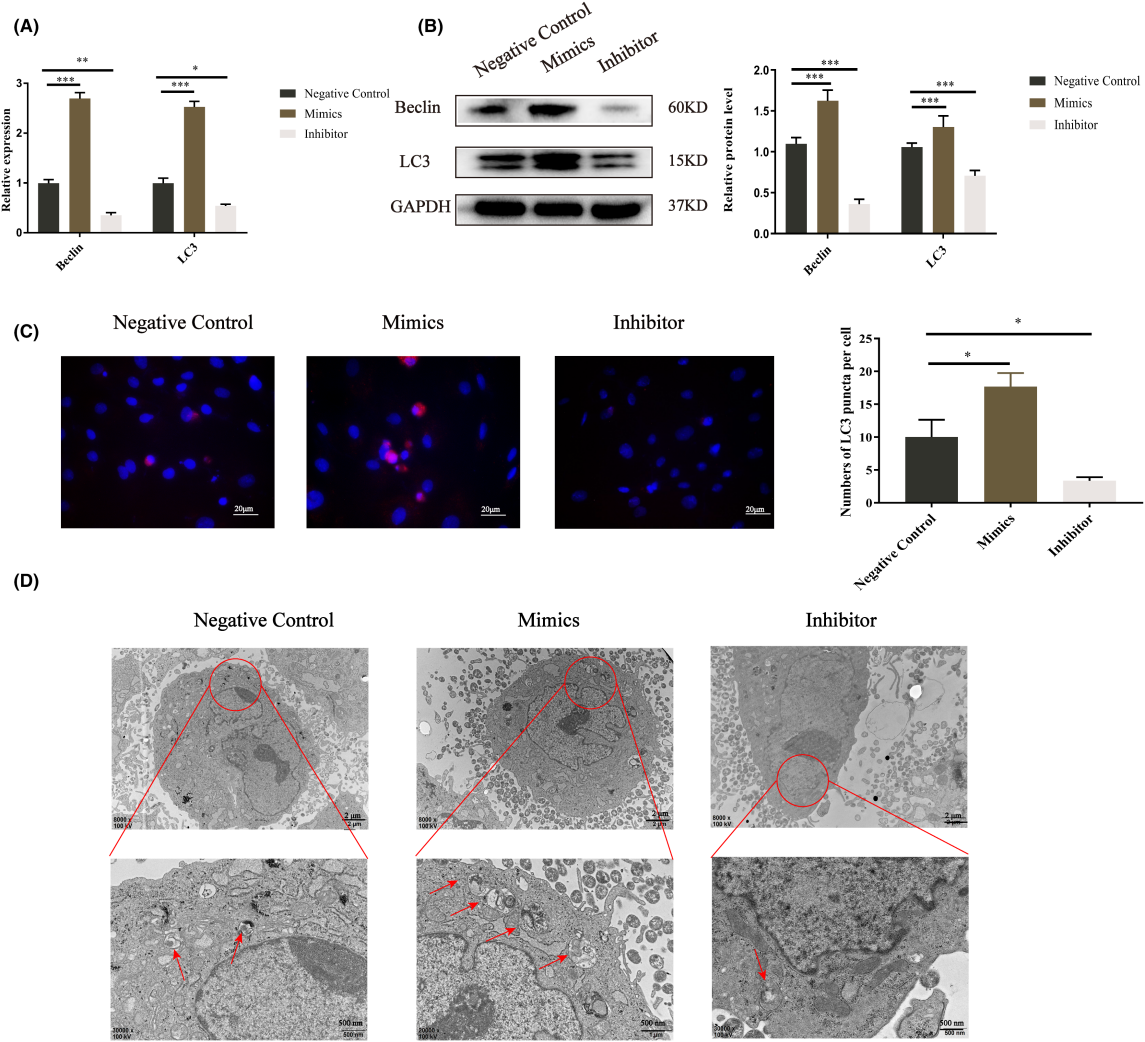
Comparison of autophagy of BMMSCs of each let‐7a group. (A) Relative mRNA levels of *Beclin* and *LC3* in BMMSCs transfected with let‐7a mimics, inhibitor and negative control. GAPDH was used for normalization. (B) Western blot of Beclin and LC3 protein level in BMMSCs transfected with let‐7a mimics, inhibitor and negative control. GAPDH was used as the internal control. (C) BMMSCs under let‐7a mimics, inhibitor and negative control treatment were subjected to immunofluorescence (IF) staining (scale bar = 20 μm); LC3 puncta: red; cell nuclei: blue. (D) Transmission electron microscopy (TEM) was used to detect autophagosomes of each group of BMMSCs (scale bar = 2 μm or 500 nm); red arrows: autophagosome. (Data are presented as means ± SEM, *n* = 3 independent experiments. *p < 0.05, **p < 0.01, ***p < 0.001).

**FIGURE 4:**
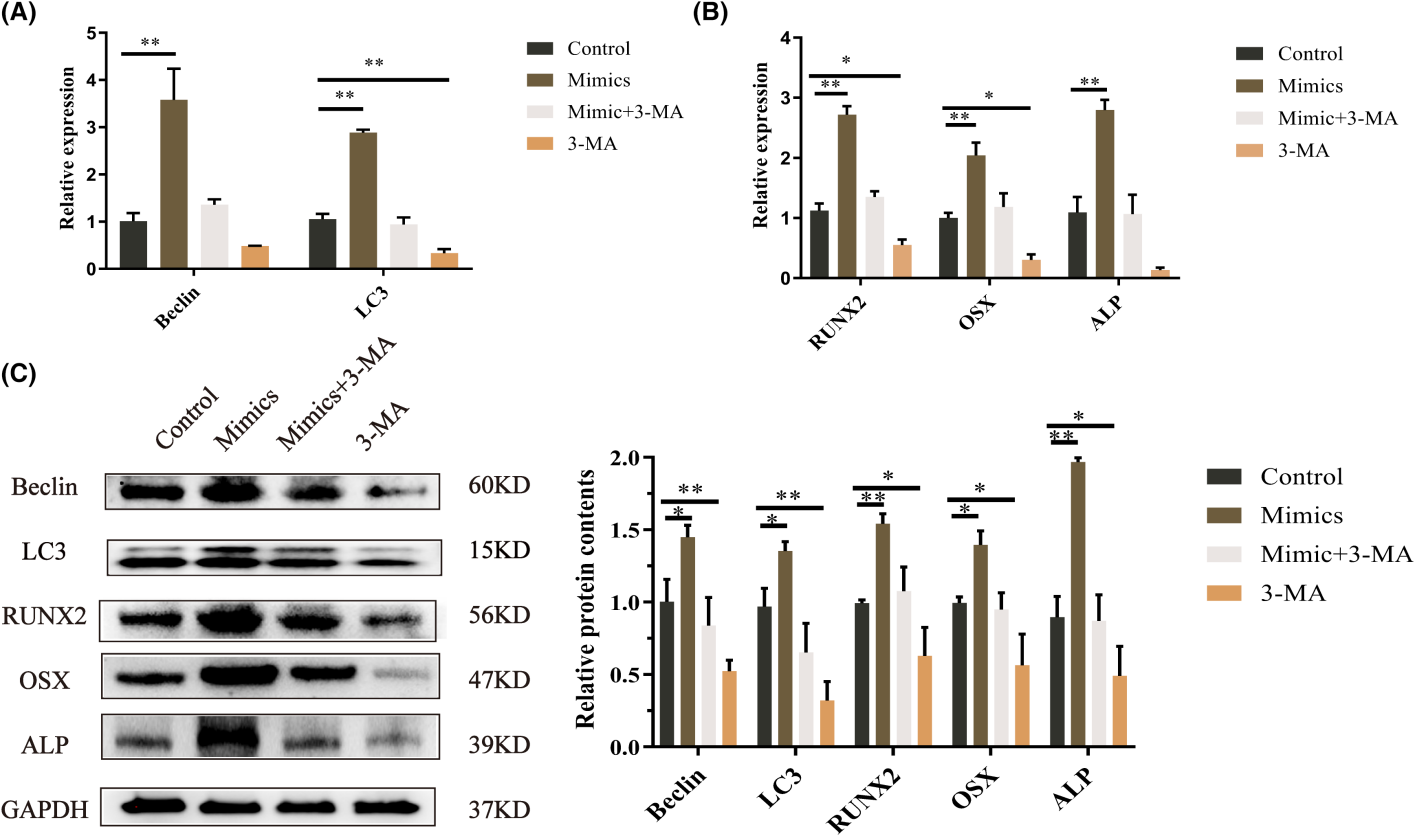
The effects of 3‐MA on the osteogenesis of BMMSCs transfected with let‐7a. (A, B) Relative mRNA levels of *Beclin* and *LC3*; *ALP*, *RUNX2* and *OSX* in BMMSCs treated by let‐7a mimics, 3‐MA or let‐7a mimics +3‐MA. GAPDH was used for normalization. (C) Western blot of Beclin and LC3; ALP, RUNX2 and OSX protein level in BMMSCs treated by let‐7a mimics, 3‐MA or let‐7a mimics +3‐MA and quantification of band intensities. GAPDH was used as the internal control. (Data are presented as means ± SEM, *n* = 3 independent experiments. *p < 0.05, **p < 0.01, ***p < 0.001).

We examined whether overexpression of let‐7a promotes BMMSC autophagy. Because autophagy is associated with Fas/FasL,^28^, ^29^ and autophagy could regulate osteogenesis.^30^ The findings indicate that Beclin and LC3 expression increased in the let‐7a mimics group but decreased in the let‐7a inhibitor group compared to the NC group (Figure 3A,B). Then, we investigated the levels of LC3 puncta in BMMSCs after transfection by treating the cells with CQ. The immunofluorescence findings demonstrated that the let‐7a mimics group accumulated more LC3 puncta than the NC group, while the let‐7a inhibitor group accumulated fewer LC3 puncta (Figure 3C). TEM was used to detect autophagosomes. The results showed the same trend as the LC3 puncta (Figure 3D). Thus, we concluded that the overexpression of let‐7a promotes autophagy in BMMSCs. We used the autophagy inhibitor 3‐MA to elucidate the connection between autophagy and the osteogenesis in BMMSCs. The results from RT‐qPCR and western blotting demonstrated that 3‐MA could significantly downregulate the gene and protein expression of autophagy‐related genes and ALP, Runx2 and OSX; however, 3‐MA plus let‐7a mimics abolished this inhibitory effect (Figure 4A–C).

### 3.4 Let‐7a promotes BMMSC osteogenesis via the Fas/FasL‐autophagy pathway

**FIGURE 5:**
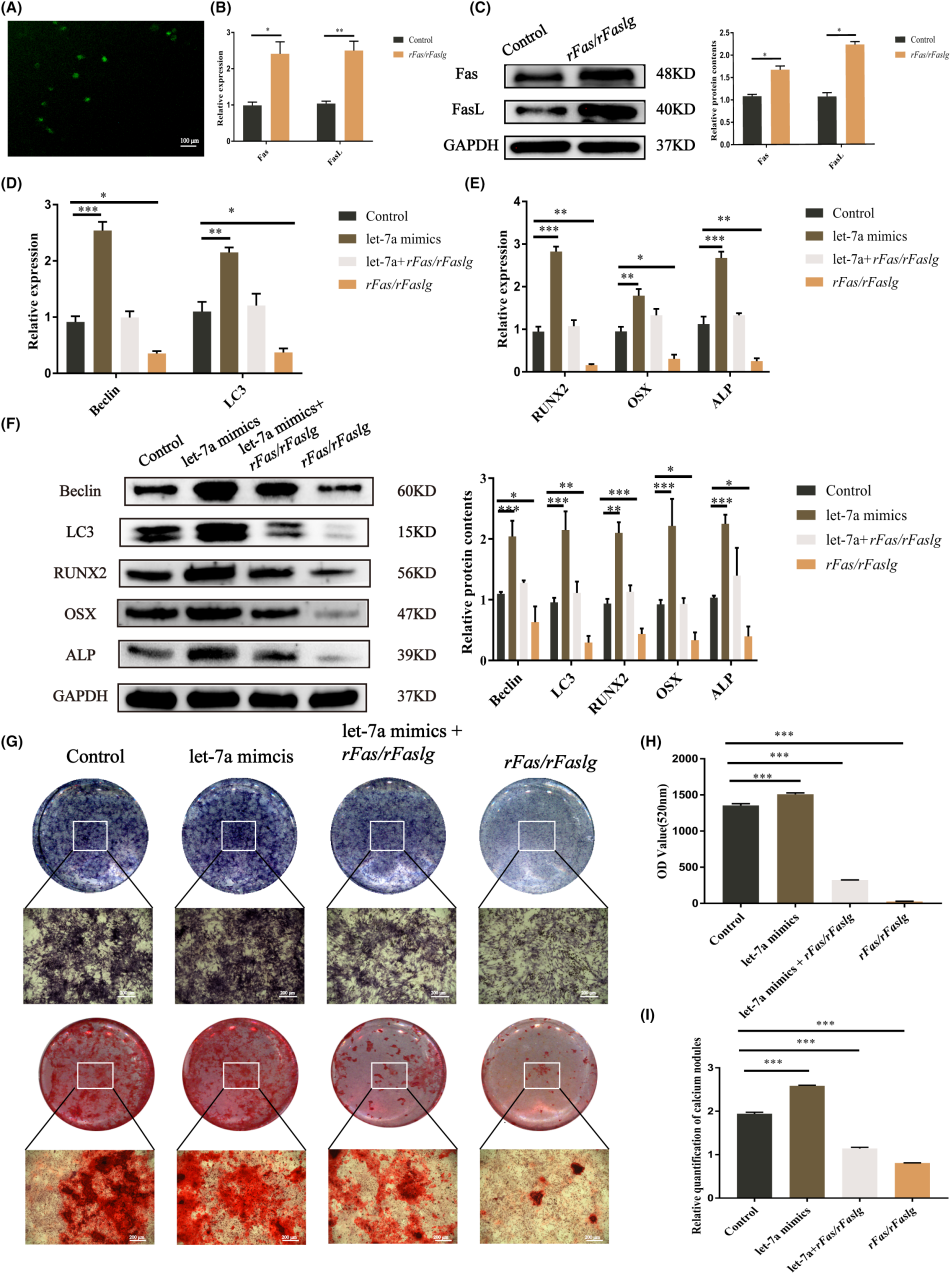
Changes in the expression of related genes and proteins after overexpression of Fas/FasL by *rFas*/*rFaslg* plasmid transfection. (A) GFP fluorescence (green) showed that plasmid was transfected into BMMSCs (scale bar = 100 μm). (B) Relative mRNA levels of *Fas* and *FasL* in BMMSCs after transfection of the *rFas*/*rFaslg* plasmid. GAPDH was used for normalization. (C) Western blot of Fas and FasL protein level in BMMSCs after transfection of the *rFas*/*rFaslg* plasmid. and quantification of band intensities. GAPDH was used as the internal control. (D, E) Relative mRNA levels of *Beclin* and *LC3*; *ALP*, *RUNX2* and *OSX* in BMMSCs treated by let‐7a mimics, *rFas*/*rFaslg* or let‐7a mimics + *rFas*/*rFaslg*. (F) Western blot of Beclin and LC3; ALP, RUNX2 and OSX protein level in BMMSCs treated by let‐7a mimics, *rFas*/*rFaslg* or let‐7a mimics + *rFas*/*rFaslg* and quantification of band intensities. GAPDH was used as the internal control. (G) ALP staining and Alizarin Red S staining of each group (control, let‐7a mimics, *rFas*/*rFaslg* or let‐7a mimics + *rFas*/*rFaslg* group) (H, I) ALP activity and quantitative comparison of mineralized nodule formation of BMMSCs of each group after let‐7a mimics, *rFas*/*rFaslg* plasmid or let‐7a mimics + *rFas*/*rFaslg* plasmid treatment. (Data are presented as means ± SEM, *n* = 3 independent experiments. *p < 0.05, **p < 0.01, ***p < 0.001).

Our previous studies demonstrated that let‐7a could directly binds Fas and FasL mRNA and suppress their expression.^17^ We also demonstrated that let‐7a promoted the osteogenesis of BMMSCs and was related to autophagy. Therefore, we tried to investigate the relationship between Fas/FasL and autophagy and then clarify whether let‐7a promotes osteogenesis via the Fas/FasL ‐autophagy pathway. GFP fluorescence showed that the *rfas*/*rfaslg* plasmid had been successfully transfected into BMMSCs (Figure 5A). In the *rFas/rFaslg* group, the levels of the Fas/FasL gene and protein were higher than those in the control group (Figure 5B–D). According to RT‐qPCR data, *rFas*/*rFaslg* plasmid transfection in BMMSCs decreased the expression of genes associated with autophagy (*Beclin* and *LC3*) and osteogenesis (*ALP*, *RUNX2*, *OSX*). The results of the western blotting matched those of the gene expression analysis (Figure 5E). According to ALP staining, ALP activity, and Alizarin Red staining, BMMSCs from the *rFas*/*rFaslg* group had a reduced ability for osteogenic differentiation (Figure 5F–I). However, the addition of let‐7a mimics counteracted this inhibitory effect. In summary, the overexpression of let‐7a promoted BMMSC osteogenesis via the Fas/FasL‐autophagy pathway (Figures S3 and S4).

## 4 DISCUSSION

Let‐7a is involved in various biological processes,^34^ but its role during osteogenesis needs further exploration. Thus, in this study, to elucidate whether let‐7a promotes the osteogenesis of BMMSCs, we overexpressed let‐7a by specific mimics in BMMSCs, and the findings demonstrated that let‐7a upregulation increased BMMSC osteogenesis in vitro (Figure 1). Bone tissue regeneration based on the osteogenesis of BMMSCs is a promising approach.^1^ However, many limitations have been found when single‐cell suspensions of BMMSCs were directly injected into the defect site, which would damage the junctions between BMMSCs. As an alternative method, CAs mainly rely on the formation of intercellular junctions and the secretion of ECM proteins to maintain cell–cell junctions and reduce cell loss and damage.^35^, ^36^ The ECM contains a variety of cytokines that can promote the formation of new bone.^37^ Moreover, because stem cells are delivered to the defective site together with the ECM, the viability and function of the cells can be maintained for a long time, and tissue regeneration can be enhanced.^38^ Several studies have also verified that CAs have been used for the treatment of periodontitis in animal models with bone defects.^32^ In addition, scaffolds are very important in tissue regeneration engineering because they can allow stem cells to survive and provide a location for them to do so.^39^ HA‐TCP is a three‐dimensional scaffold commonly used in bone tissue regeneration engineering that is highly biocompatible and may promote stem cell osteogenesis.^40^ Several research groups have reported the use of MSC‐CAs plus HA‐TCP for periodontal bone regeneration. Thus, in this study, HA‐TCP was used as the scaffold material for BMMSC‐CAs transfected with let‐7a to implant into a rat model of a periodontal bone lesion, and the results demonstrated that let‐7a promoted the bone defect reconstructive capacity of the BMMSC‐CA/HA‐TCP complex (Figure 2).

A previous study found that let‐7 positively influences stem cell osteogenesis^41^; however, more research is needed to determine the precise functions of miRNA‐let‐7a in osteogenesis as well as the underlying molecular pathways. Ma et al. discovered that let‐7a inhibited BMMSC osteogenesis in mice with postmenopausal osteoporosis (PMOP) by regulating TGFBR1 expression.^42^ In contrast, our results showed that let‐7a promotes the osteogenesis of BMMSCs. The discrepancy may be due to differences in the BMMSCs employed in the two studies. Notably, TGF‐β1 in BMMSCs obtained from normal rats was not affected; therefore, the downregulation of TGF‐β1 by let‐7a did not decrease the osteogenesis of BMMSCs that we cultured. Another study showed that let‐7a upregulation can reduce RNA KCNQ1OT1 and promote osteoblast differentiation,^43^ and this result aligned closely with our study results.

Previous research has shown that let‐7a can inhibit the Fas/FasL expression.^17^ To ascertain the fundamental processes through which let‐7a promotes BMMSC osteogenesis, we constructed a plasmid carrying *rfas*/*rfaslg* to overexpress Fas/FasL for BMMSC transfection. The results of RT‐qPCR and western blotting showed that Fas and FasL expression was increased and that the osteogenesis‐associated genes *OSX*, *RUNX2* and *ALP* were downregulated (Figure 5). This finding is consistent with a previous study, which demonstrated that activation of Fas by its ligand FasL negatively regulates the osteogenic differentiation of osteoblasts from bone marrow.^44^ Furthermore, we demonstrated that let‐7a mimics alleviated the inhibited osteogenesis of BMMSCs by the *rfas*/*rfaslg* plasmid (Figure 5). Because upregulation of let‐7a and downregulation of Fas/FasL, as well as their targeted relationship, may promote the osteogenesis of BMMSCs, let‐7a regulation of the osteogenesis of BMMSCs may be realized through the Fas/FasL pathway.

As a classic apoptosis pathway,^45^ Fas/FasL is associated with autophagy.^28^ For example, Fas/FasL activates autophagy by targeting the Fas‐activated death domain (FADD), Src, members of the c‐Jun N‐terminal kinase (JNK) family of stress kinases, BECN1, PI3K,^46^, ^47^, ^48^, ^49^ etc. Furthermore, the autophagy inhibitor 3‐MA could obviously reverse the outcomes regulated by Fas/FasL signalling.^46^, ^47^, ^48^, ^49^ Accordingly, our study illustrated that overexpression of Fas and FasL downregulated autophagy in BMMSCs (Figure 5). Additionally, autophagy is widely recognized as a crucial element in osteogenesis.^50^ Ma and colleagues demonstrated that autophagic suppression inhibited osteogenesis while promoting adipogenesis in BMMSCs.^33^ Our study also demonstrated that the autophagy inhibitor 3‐MA reduced the osteogenesis of BMMSCs; moreover, let‐7a mimics alleviated the inhibited osteogenic differentiation of BMMSCs by 3‐MA (Figure 4). Furthermore, the autophagy rate was increased in BMMSCs after let‐7a was overexpressed (Figure 3). Therefore, we concluded that let‐7a could regulate the osteogenesis of BMMSCs via the Fas/FasL‐autophagy pathway.

In summary, the interaction of Fas/FasL and let‐7a affects both osteogenic differentiation and autophagy in BMMSCs. Based on the negative correlation between Fas/FasL and let‐7a^17^ and the relationship between Fas/FasL and autophagy as previously reported,^28^ we studied their crosstalk and investigated their effects on osteogenesis in BMMSCs. However, whether let‐7a affects the osteogenesis of BMMSCs by targeting other molecules in addition to Fas/FasL remains unclear. Studies have shown that let‐7a can target IGF1R, mitogen‐activated protein kinase (MAPK), and TGF‐β1^51^ and suppress their expression, which may influence BMMSC osteogenesis directly or indirectly. Furthermore, let‐7a could accelerate autophagy by targeting STAT3,^52^ BCL‐xL^53^ or NUAK1.^54^ Therefore, the notion that let‐7a stimulates BMMSC osteogenesis via mechanisms other than Fas/FasL cannot therefore be ruled out and further investigation is needed.

The present study demonstrates that let‐7a overexpression inhibited the Fas/FasL expression and then activated autophagy, finally promoting the osteogenesis of BMMSCs and enhancing the bone regenerative effects in a periodontal defect model of BMMSCs‐CAs. The findings highlight the application of let‐7a in BMMSC‐CAs as a possible periodontal bone regeneration strategy in the future.

## 5 CONCLUSION

In conclusion, our research reveals that let‐7a can effectively enhance the osteogenesis of rat BMMSCs via the Fas/FasL‐autophagy pathway and can improve the bone defect reconstructive capacity of BMMSC‐CAs. These findings indicate that let‐7a‐overexpressing BMMSC‐CAs can be a possible therapeutic strategy to regenerate periodontal bone defects in future clinical applications.

## AUTHOR CONTRIBUTIONS

**Shiyao Yang:** Conceptualization (equal); data curation (equal); formal analysis (equal); investigation (equal); methodology (equal); resources (lead); software (equal); writing – original draft (equal); writing – review and editing (equal). **Jing Gao:** Conceptualization (equal); data curation (equal); formal analysis (equal); investigation (equal); project administration (equal); writing – original draft (equal). **Meng Chen:** Conceptualization (supporting); data curation (supporting); investigation (supporting); methodology (supporting); software (supporting). **Yuting Sun:** Methodology (supporting); software (supporting). **Xin Qiao:** Methodology (supporting); software (supporting). **Hongchen Mao:** Methodology (supporting); software (supporting). **Li Guo:** Methodology (supporting); software (supporting). **Yang Yu:** Conceptualization (lead); investigation (lead); methodology (lead); project administration (lead); supervision (lead); writing – review and editing (lead). **Deqin Yang:** Conceptualization (lead); funding acquisition (lead); project administration (lead); supervision (lead); writing – review and editing (lead).

## CONFLICT OF INTEREST STATEMENT

The authors have declared that no competing interest exists.

## Supporting information


Figure S1.

## Data Availability

The data that support the findings of this study are available from the corresponding author upon reasonable request.
