# Supplementary material for: Let‐7a promotes periodontal bone regeneration of bone marrow mesenchymal stem cell aggregates via the Fas/FasL‐autophagy pathway
Source: J Cell Mol Med. 2023 Oct 19;27(24):4056–68. doi: 10.1111/jcmm.17988 (PMC10746947; doi:10.1111/jcmm.17988)
Supplement: Supplementary file 1 — Figure S1. [file JCMM-27-4056-s001.docx]

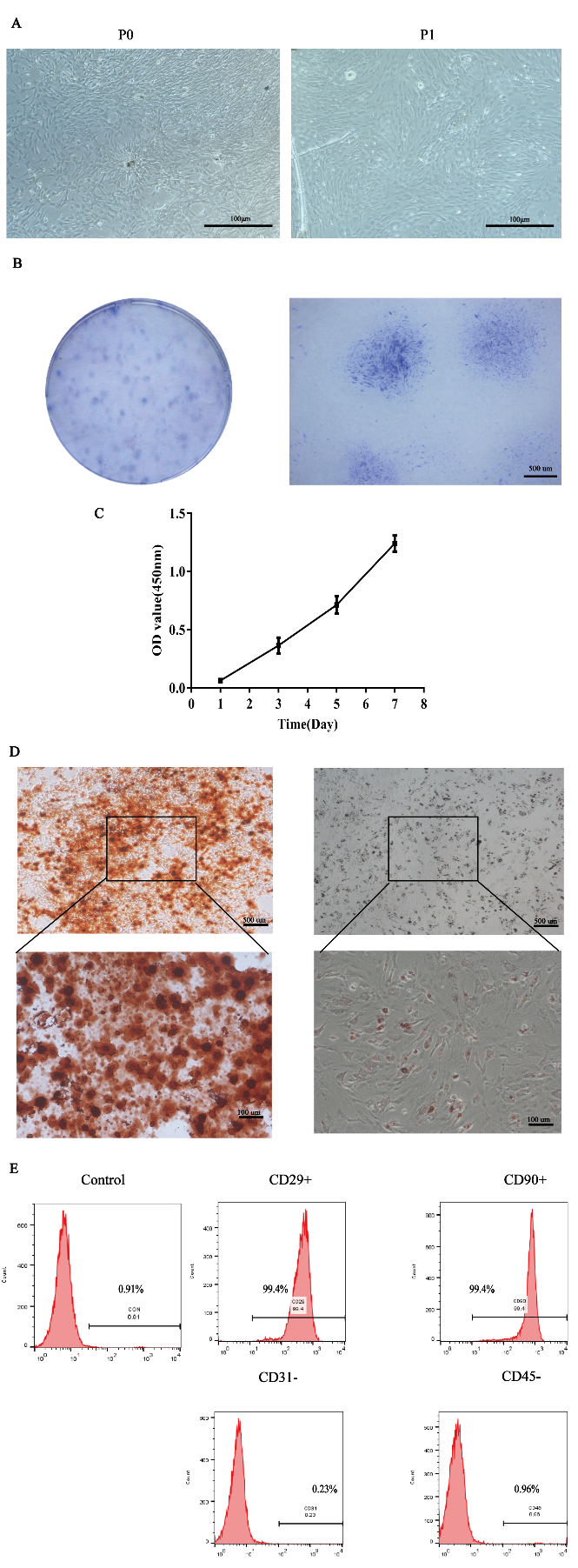


Figure S1. Isolation and characterization of bone marrow mesenchymal stem cells (BMMSCs). (A) Representative images of long spindle-shaped morphology by BMMSCs of primary and P1 after passage (scale bar = 50 μm). (B) Identification of cloning-forming ability of BMMSCs (scale bar = 500 μm). (C) Growth curves of BMMSCs determined by CCK8 assay. (D) Alizarin Red S and Oil Red O staining of BMMSCs (scale bar = 500 μm or 100 μm). (E) Flow cytometric analysis of BMMSCs.


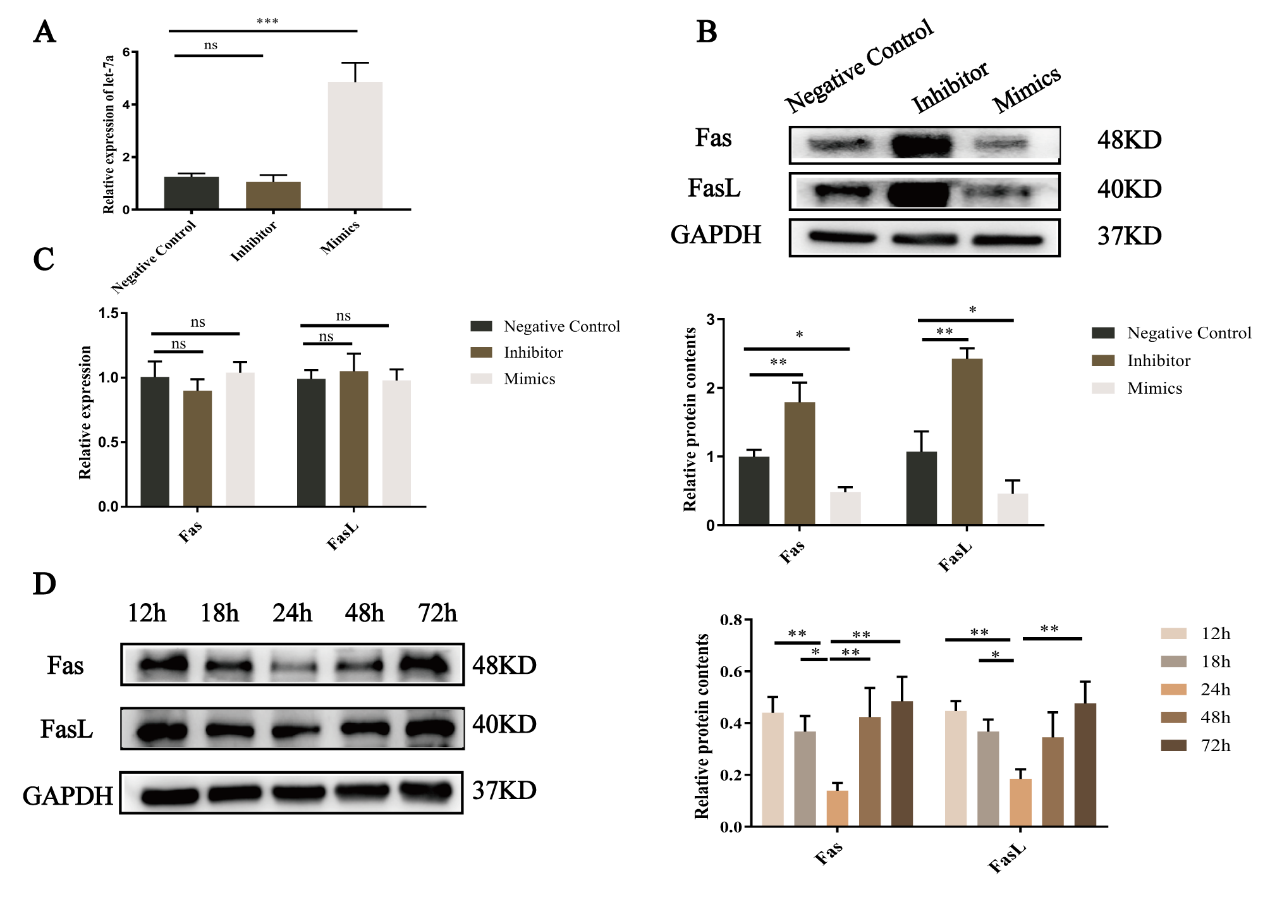


Figure S2. Let-7a transfection inhibits the expression of Fas and FasL. (A) Relative expression of let-7a after let-7a mimics, inhibitor and negative control transfection. (B) Relative mRNA levels of *Fas* and *FasL* in BMMSCs after let-7a mimics, inhibitor and negative control transfection. *GAPDH* was used for normalization. (C) Western blot of Fas and FasL protein level in BMMSCs after let-7a mimics, inhibitor and negative control transfection and quantification of band intensities. GAPDH was used as the internal control. (D) The expression of Fas and FasL protein in BMMSCs at 12h, 18h, 24h,48h and 72h after let-7a mimics transfection. (Data are presented as means ± SEM, n = 3 independent experiments. *p < 0.05, **p < 0.01, ***p<0.001)


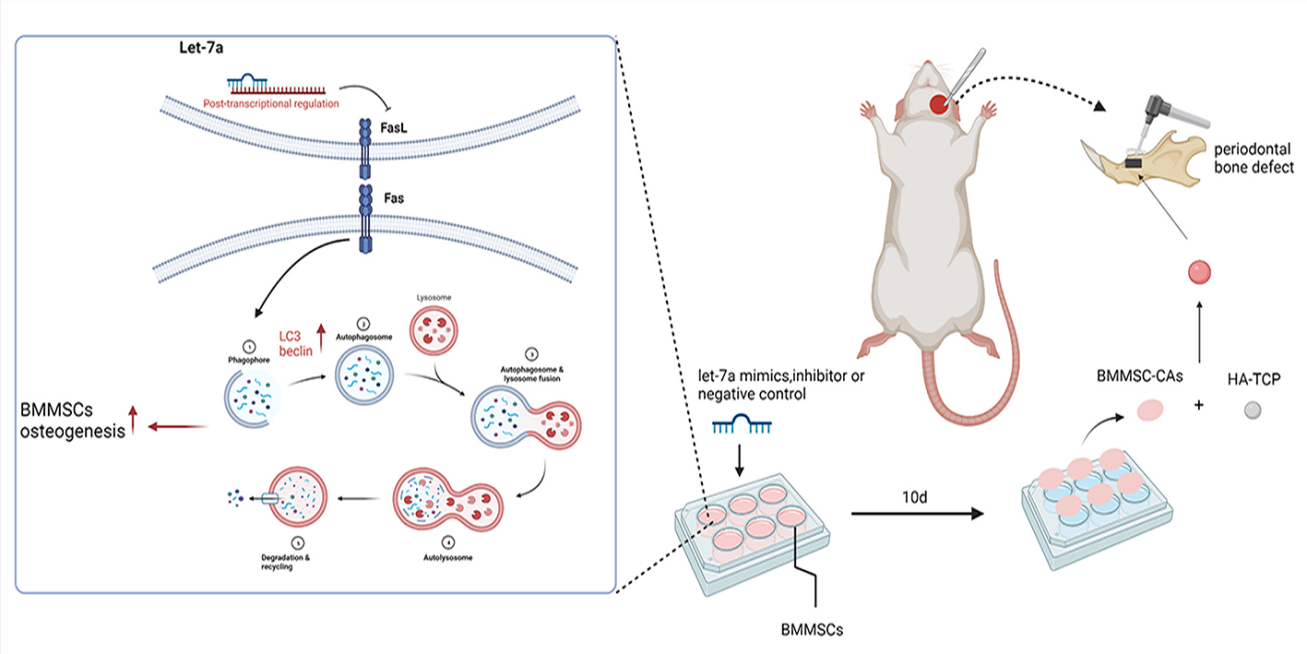


Figure S3. The experimental process of let-7a transfected BMMSC-CAs in periodontal bone regeneration.


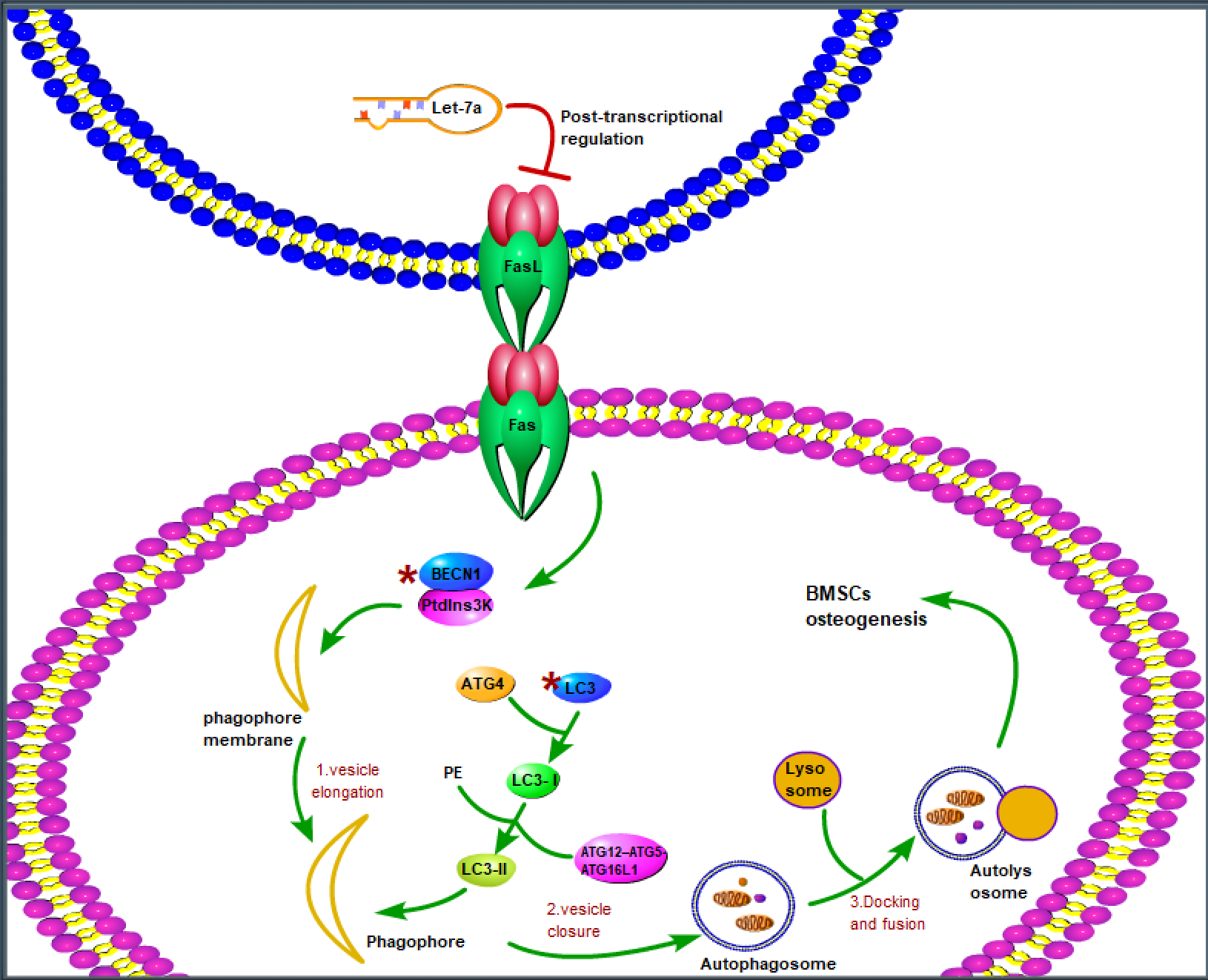


Figure S4. The proposed mechanism model for the effect of let-7a on the osteogenesis of BMMSCs.
